# Tax contributes apoptosis resistance to HTLV-1-infected T cells via suppression of Bid and Bim expression

**DOI:** 10.1038/cddis.2014.536

**Published:** 2014-12-18

**Authors:** A Mühleisen, M Giaisi, R Köhler, P H Krammer, M Li-Weber

**Affiliations:** 1 Tumor Immunology Program D030, German Cancer Research Center (DKFZ), Heidelberg 69120, Germany

## Abstract

The human T-lymphotropic virus type 1 (HTLV-1) is the etiological agent of adult T-cell leukemia (ATL). HTLV-1 Tax has been shown to have a prosurvival role in infected T cells by enhancing expression of the Bcl-2 family of antiapoptotic proteins. In this study, we show that the expression of proapoptotic BH3-only proteins Bim (Bcl-2-interacting mediator of cell death) and Bid (BH3-interacting domain death agonist) is diminished in HTLV-1-infected leukemic cells. Using a Tax-inducible system and a transient overexpression approach, we demonstrate that Tax downregulates Bid and Bim expression at the transcriptional level. We show that reinforced expression of Bim and Bid in HTLV-1-infected T-cell lines sensitizes CD95/TRAIL- and anticancer drug-induced apoptosis. Furthermore, we show that Tax suppresses Bid and Bim expression by enhancing hypoxia-inducible factor-1*α* (HIF-1*α*) protein expression. siRNA knockdown of HIF-1*α* or chemical inhibition of the transactivation activity of HIF-1*α* resulted in an increase in Bid and Bim expression and, consequently, in an increase in CD95/TRAIL- and anticancer drug-induced apoptosis in HTLV-1-infected leukemic T-cell lines. Our study provides evidence that besides upregulation of prosurvival Bcl-2 proteins, Tax may also confer apoptosis resistance to HTLV-1-infected T cells by suppressing the expression of the proapoptotic BH3-only proteins Bim and Bid.

The adult T-cell leukemia (ATL) was first described in 1977. Hereafter, the exclusive causal agent for ATL was identified to be a retrovirus, the human T-lymphotropic virus type 1 (HTLV-1), in 1980.^1^ At present, an estimated 10 million people worldwide are infected with HTLV-1.^2^ Although only a small portion of virus carriers (~6.6% for males and 2.1% for females) will develop ATL, those patients have a poor prognosis with a survival range of <1 year after disease onset.^3, 4^

After more than 30 years of intensive studies, evidence has shown that the viral protein Tax has a key role in promoting viral spread and it is also one of the essential proteins involved in oncogenesis through multiple mechanisms, for example, promoting G1–S progression, enhancing the PI3K-AKT signaling pathway, inducing DNA hyper-replication, decreasing DNA repair, constitutive activation of NF-*κ*B and suppression of apoptosis.^4, 5^ We and others have previously shown that HTLV-1 Tax enhances expression of the caspase-8 inhibitory protein c-FLIP and the Bcl-2 family of antiapoptotic proteins Bcl-2, Bcl-xL, Mcl-1 and Blf-1, and, consequently, causes apoptosis resistance and survival of HTLV-1-infected T cells.^6, 7, 8, 9, 10, 11^ So far, most studies focused on the effect of Tax on antiapoptotic proteins. Little is known about the influence of HTLV-1 Tax on proapoptotic proteins. As pro- and antiapoptotic proteins are equally important in the regulation of death and life of cells, we asked whether Tax-mediated apoptosis resistance and survival also involves deregulation of proapoptotic genes.

To date, the best studied mammalian proapoptotic proteins are the apoptotic effector proteins Bak (Bcl-2 antagonist/killer) and Bax (Bcl-2-associated X protein), which contain three BH domains (BH1, BH2 and BH3)^12, 13^ and the BH3-only proteins Bad (Bcl-2 antagonist of cell death), Bid (BH3-interacting domain death agonist), Bim (Bcl-2-interacting mediator of cell death), Noxa and PUMA (p53-upregulated modulator of apoptosis).^14, 15^ Cell death induced by BH3-only proteins is absolutely dependent on the presence of Bak and Bax, indicating that they act upstream of these effector proteins in the apoptotic signaling pathway.^14, 15^ Functionally, BH3-only proteins can be divided into activator and sensitizer proteins depending on their ability to interact with other members of the Bcl-2 family. The activator BH3-only proteins Bid, Bim and PUMA bind not only to all antiapoptotic Bcl-2 members but are also capable of activating Bak and Bax directly.^16, 17, 18, 19, 20^ The sensitizer BH3-only proteins, however, only interact with a limited set of antiapoptotic proteins, for example, Bad binds to Bcl-2, Bcl-xL and Bcl-w, whereas Noxa exclusively interacts with Mcl-1 and A1.^17^

In this study, we show that Bid and Bim expression is suppressed in HTLV-1-infected leukemic T-cell lines. We also show that Tax downregulates Bid and Bim expression through the elevation of the protein levels of the transcription factor HIF-1*α* (hypoxia-inducible factor-1*α*). Furthermore, we show that suppression of Bid and Bim expression by Tax may have a significant impact on apoptosis resistance to CD95, TRAIL-R stimulation and anticancer drugs in HTLV-1-infected T cells.

## Results

### HTLV-1-infected T cells are highly resistant to anticancer drug-induced apoptosis

**Figure 1:**
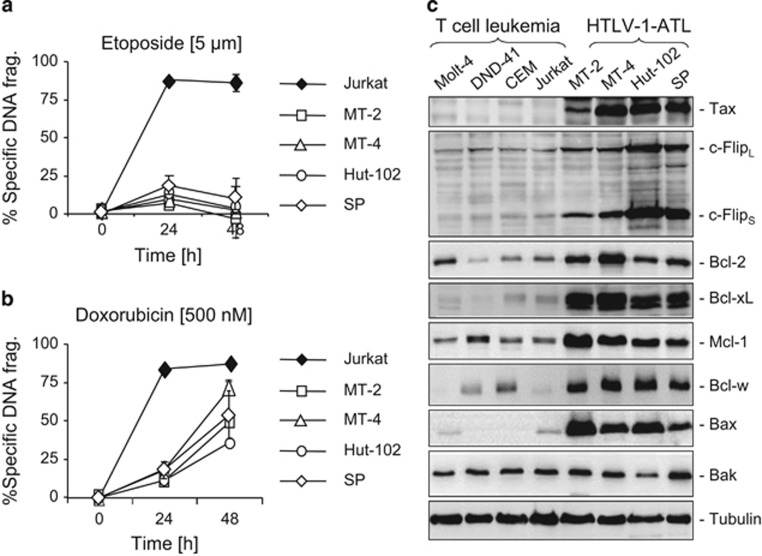
HTLV-1-infected T cells are highly resistant to anticancer drugs and show altered expression profiles of pro- and antiapoptotic proteins. (**a** and **b**) HTLV-1-infected T cells are resistant to etoposide- and doxorubicin-induced cell death. Cells from four HTLV-1-infected cell lines, MT-2, MT-4, Hut-102 and SP, were treated with etoposide (5 *μ*M) or doxorubicin (500 nM) for 24 and 48 h. Apoptosis was determined by DNA fragmentation. The non-HTLV-1-infected leukemic T-cell line Jurkat served as a control. Results are representative of three independent experiments each performed in duplicate assays. (**c**) Comparison of expression levels of pro- and antiapoptotic proteins between the HTLV-1-infected T-cell lines and the non-HTLV-1-infected leukemic T-cell lines. Total cell lysates from MT-2, MT-4, Hut-102 and SP and the non-HTLV-1-infected leukemic T-cell lines Molt-4, DND-41, CEM and Jurkat were subjected to western blot analysis by antibodies against different anti- and proapoptotic proteins as indicated. Tax protein expression is used as a control of HTLV-1 infection. Representative blots from two to three independent experiments are shown

Up to date, clinical trials on ATL with even a wide range of combination chemotherapy achieved only limited success.^3^ To investigate the molecular basis of their chemotherapy resistance, we chose four HTLV-1-infected T-cell lines, either derived from HTLV-1-infected ATL patients (Hut-102 and SP) or HTLV-1 transformed by coculture (MT-2 and MT-4), and tested their response to etoposide and doxorubicin treatment. Compared with the non-HTLV-1-associated leukemic T-cell line Jurkat, all four HTLV-1-infected cell lines tested were highly resistant to etoposide- and doxorubicin-induced cell death at the doses that killed >75% of Jurkat T cells (Figures 1a and b). We then carried out a systematic analysis of the expression levels of proteins involved in apoptosis regulation by western blot analysis. Consistent with previous studies,^6, 7, 8, 9, 10, 21^ all four HTLV-1-infected T-cell lines showed elevated expression levels of the antiapoptotic proteins (Figure 1c). Noticeably, the expression level of the proapoptotic effector protein Bax was also markedly increased in HTLV-1-infected T-cell lines (Figure 1c). As Bax is essential for apoptosis induction,^15^ the observation above raises the question how HTLV-1 confers apoptosis resistance to infected cells when the expression levels of both anti- and proapoptotic proteins are enhanced.

### Bid and Bim expression is downregulated in HTLV-1-infected T cells

**Figure 2:**
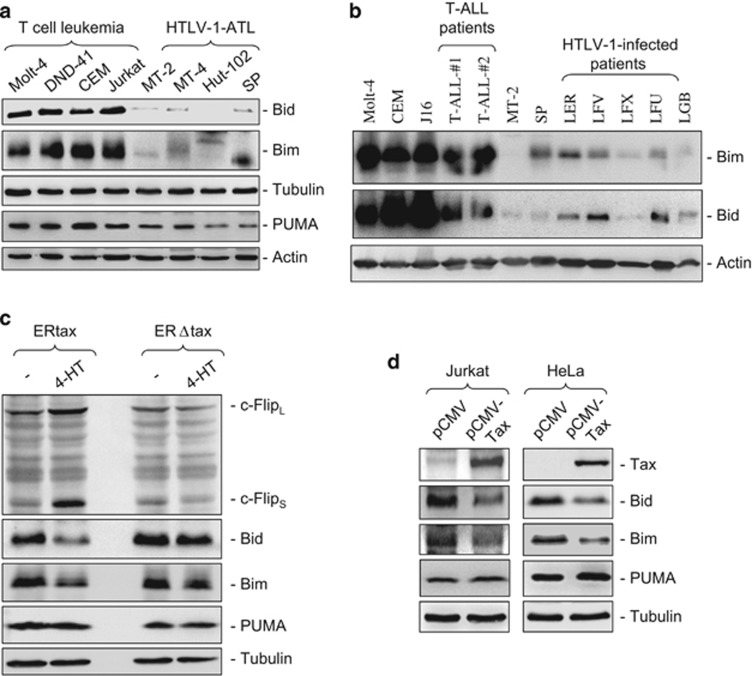
Bim and Bid expression is suppressed in HTLV-1-infected T cells. (**a**) Bim and Bid expression is suppressed in HTLV-1-infected T-cell lines. Total cell lysates from indicated HTLV-1-Tax-positive T-cell lines and non-HTLV-1-infected leukemic T-cell lines were subjected to western blot analysis by antibodies against Bim, Bid and PUMA. Representative blots from three independent experiments are shown. (**b**) Bim and Bid expression is suppressed in primary HTLV-1-positive ATL cells. Cells from HTLV-1-positive ATL patients were subjected to western blot analysis for Bim and Bid as in (**a**). The non-HTLV-1-infected cell lines Molt-4, CEM, Jurkat, the HTLV-1-infected MT-2 and SP cell lines and two T-ALL patients were used as controls. Representative blots from two independent experiments are shown. (**c** and **d**) Downregulation of Bim and Bid expression correlates with Tax expression. Jurkat cells stably transfected with ERtax or ERΔtax fusion proteins were stimulated by 4-TH (5 *μ*M) for 40 h and then subjected to western blot analysis with antibodies as indicated (**b**). As ERtax and ERΔtax fusion proteins do not react with anti-Tax antibodies, c-Flip expression levels were used as positive controls for Tax function. Representative blots from three independent experiments are shown. (**c**) Jurkat and HeLa cells were transiently transfected with a Tax expression plasmid pCMV-Tax. Twenty-four hours after transfection, total cell lysates were subjected to western blot analysis with antibodies as indicated. Representative blots from two (Jurkat) and three (HeLa) independent experiments are shown

It has been shown that Bax activity can be directly enhanced by Bim, Bid and PUMA.^18, 22, 23^ Therefore, we asked whether the expression levels of Bim, Bid and PUMA are also altered to avoid an increase in apoptosis due to increased Bax expression. To address this question, we examined the expression levels of Bim, Bid and PUMA in HTLV-1-infected T-cell lines. Interestingly, we found that Bim and Bid expression levels were markedly downregulated in all HTLV-1-infected T-cell lines tested (Figure 2a). To confirm this observation, primary leukemic cells from two T-ALL patients and five HTLV-1-infected ATL patients were subjected to the analysis of Bim and Bid expression. Consistent with the observation from the ATL cell lines, primary ATL cells also showed a significantly reduced expression of Bim and Bid compared with the non-HTLV-1-infected T-ALL cells (Figure 2b).

We then asked whether Tax is responsible for the reduction of Bid, Bim and PUMA expression. To address this question, we first used an inducible system in which Jurkat cells were stably transfected with an estrogen receptor-tax fusion protein (ERtax) or an estrogen receptor-truncated tax (lacking the first 12 amino acids) fusion protein (ERΔtax).^24^ ERtax can be activated by exogenous addition of 4-hydroxytamoxifen (4-HT). Using this inducible system, we observed that activation of ERtax but not ERΔtax resulted in the suppression of Bim and Bid expression (Figure 2c). Consistent with our previous studies,^8^ the expression levels of c-FLIP proteins were enhanced by ERtax and served as a positive control. Next, to confirm this result, we carried out a transient transfection experiment using an expression plasmid to express Tax in either Jurkat or HeLa cells. The experiments showed that ectopic expression of Tax downregulated Bim and Bid expression in Jurkat and HeLa cells (Figure 2d). In contrast, PUMA expression was not influenced by Tax. These experiments demonstrate that Tax is responsible for the reduction of Bim and Bid expression.

### Overexpression of Bid and Bim sensitizes HTLV-1 infected cells towards apoptosis

**Figure 3:**
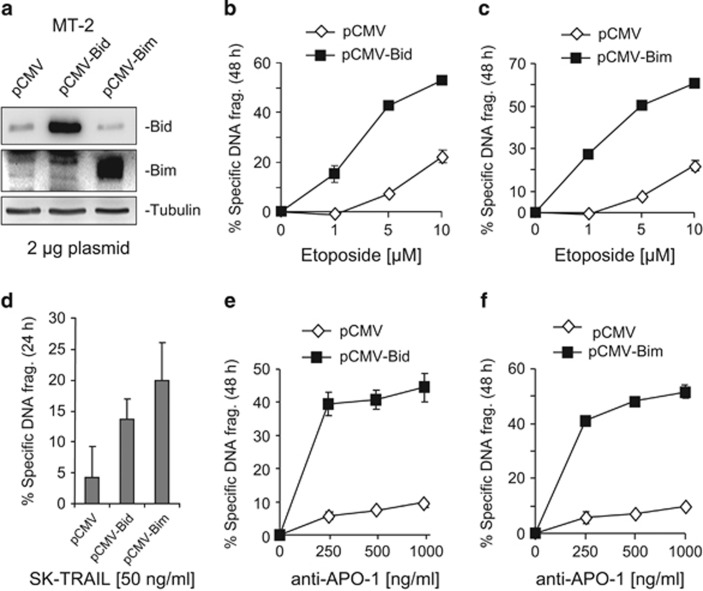
Overexpression of Bid and Bim sensitizes HTLV-1-infected T cells towards etoposide-, TRAIL- and anti-CD95-mediated apoptosis. (**a**) Overexpression of Bid and Bim in MT-2 cells. MT-2 cells were transfected with Bim or Bid expression plasmids as described in Materials and Methods. Forty-eight hours after transfection, expression efficiencies of Bim and Bid were controlled by western blot analysis. (**b** and **c**) Overexpressions of Bid and Bim sensitize HTLV-1-infected T cells towards etoposide-induced apoptosis. Forty-eight hours after transfection, MT-2 cells were treated with different concentrations of etoposide for 24 h as indicated. Apoptotic cell death was determined by DNA fragmentation. Results are representative of two independent experiments each performed in duplicate assays. (**d**) Overexpressions of Bid and Bim sensitize HTLV-1-infected T cells towards TRAIL-induced apoptosis. The experiment was performed as in (**b** and **c**). Cells were treated with 50 ng/ml TRAIL for 24 h. Results are representative of two independent experiments each performed in duplicate assays. (**e** and **f**) Overexpressions of Bid and Bim sensitize HTLV-1-infected T cells towards anti-CD95-induced apoptosis. The experiments were performed as in (**b**–**d**). Cells were treated with different concentrations of anti-APO-1 (CD95) antibody for 24 h. Apoptotic cell death was determined by DNA fragmentation. Results are representative of two independent experiments each performed in duplicate assays

To investigate the importance of Bid or Bim in the regulation of sensitivity and resistance towards apoptosis induction in HTLV-1-infected T cells, we transiently transfected expression plasmids containing either Bid or Bim cDNA into the HTLV-1-infected T-cell lines MT-2 and Hut-102. The experiments showed that ectopic expression of Bid or Bim in MT-2 and Hut-102 cells significantly enhanced the sensitivities of these cells towards etoposide-induced apoptosis (Figures 3a–c and Supplementary Figures S1A and B). HTLV-1-infected T cells have shown resistance to CD95L- and TRAIL-induced cell death.^8, 9, 25^ Consistent with the previous studies, all four HTLV-1-infected T-cell lines used in this study were highly resistant to CD95- and TRAIL-induced apoptosis (Supplementary Figure S2). Ectopic expression of Bid or Bim also enhanced CD95- and TRAIL-induced cell death in MT-2 (Figures 3d–f) and Hut-102 cells (Supplementary Figures S1C and D).

**Figure 4:**
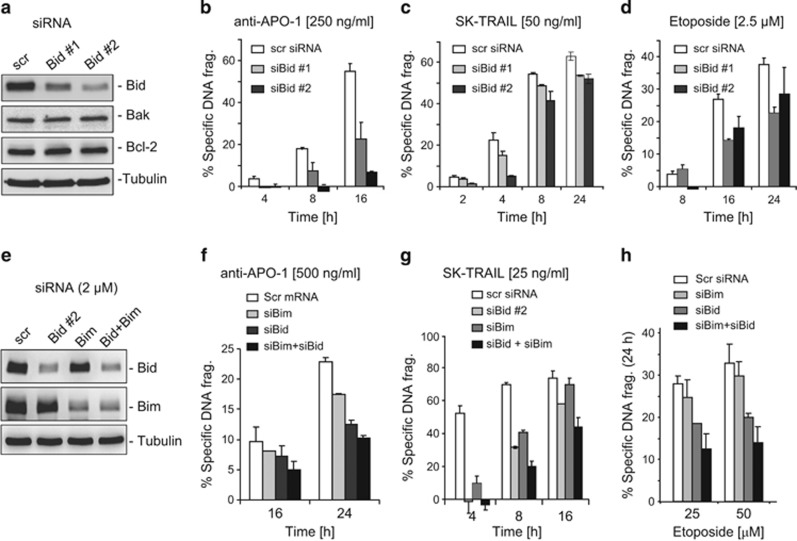
Knockdown of either Bim or Bid reduced apoptotic cell death induced by anti-CD95, TRAIL or etoposide. (**a**) Knockdown of Bid in Jurkat T cells. Jurkat T cells were transfected with two siRNAs specific for Bid. Seventy-two hours after transfection, the specificity and efficacy of knockdown were controlled by western blot with antibodies as indicated. (**b**–**d**) Knockdown of Bid reduced apoptotic cell death induced by anti-CD95, TRAIL or etoposide. Seventy-two hours after transfection, cells were treated with anti-APO-1 (CD95) (250 ng/ml), TRAIL (50 ng/ml) or etoposide (2.5 *μ*M) for different time periods as indicated. Apoptotic cell death was determined by DNA fragmentation. Results are representative of two independent experiments each performed in duplicate assays. (**e**) Knockdown of Bid, Bim or both in Jurkat T cells. Jurkat T cells were transfected with siRNAs specific against Bid or Bim. Forty-eight hours after transfection, the specificity and efficacy of knockdown were controlled by western blot with antibodies as indicated. (**f**–**h**) Knockdown of Bid, Bim or both reduced apoptotic cell death induced by anti-APO-1 (CD95), TRAIL and etoposide. The experiments were performed as in (**b** and **c**). Results are representative of three independent experiments each performed in duplicate assays

To further confirm the importance of Bim and Bid in the contribution of apoptosis sensitivity, we carried out an siRNA approach to knockdown Bim and Bid in the non-HTLV-1-infected T-cell line Jurkat. The experiments showed that knockdown of either Bim or Bid reduced apoptotic cell death induced by anti-CD95, TRAIL and etoposide (Figure 4). Knockdown of both proteins Bim and Bid further reduced anti-CD95-, TRAIL- and etoposide-induced apoptosis (Figures 4e–h). Similar results were obtained when Bid was knocked down in HeLa cells (Supplementary Figure S3). Differences in the role of Bid in the regulation of CD95- and TRAIL-induced cell death were also observed. As shown in Figures 4b and c, knockdown of Bid showed a stronger effect on CD95-mediated cell death compared with that seen in TRAIL-mediated cell death. These data demonstrate that Bim and Bid have an important role in promoting anti-CD95/TRAIL- and anticancer drug-induced cell death.

### Tax suppresses Bim and Bid expression by the upregulation of HIF-1*α* protein levels

**Figure 5:**
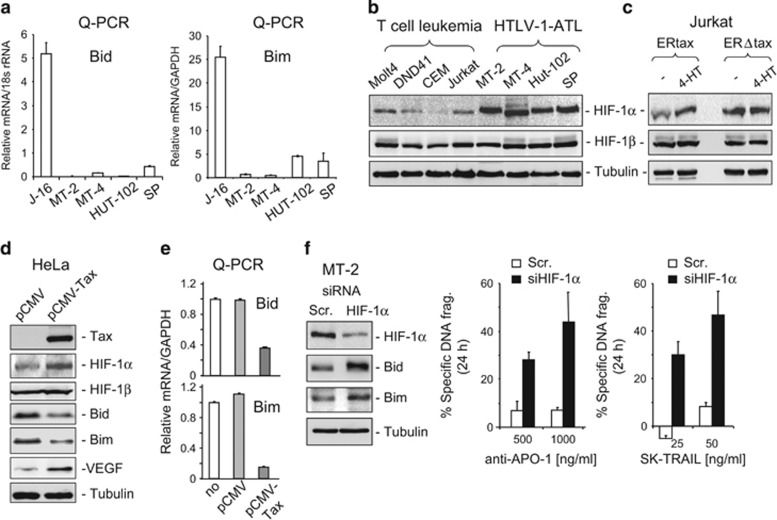
Tax suppresses Bim and Bid expression at the transcriptional level by the upregulation of HIF-1*α* protein expression. (**a**) Bid and Bim mRNA expression is downregulated in HTLV-1-Tax-expressing cells. The mRNA expression levels of Bid and Bim in MT-2, MT-4, Hut-102 and SP were compared with the non-HTLV-1-infected leukemic T-cell line Jurkat by q-PCR. Results are representative of two independent experiments each performed in triplicate assays. (**b**) HTLV-1-Tax-expressing T cells express elevated levels of HIF-1*α* proteins. Western blot analysis of the expression levels of HIF-1*α* and HIF-1*β* protein in MT-2, MT-4, Hut-102 and SP cells. Molt-4, DND-41, CEM and Jurkat cells were used as controls. Representative blots from three independent experiments are shown. (**c** and **d**). Increased HIF-1*α* protein expression correlates with Tax expression. (**c**) Jurkat ERtax or ERΔtax cells were stimulated by 4-TH (5 *μ*M) for 40 h and then subjected to western blot analysis with antibodies against HIF-1*α* and HIF-1*β* as indicated. Representative blots from two independent experiments are shown. (**d**) HeLa cells were transiently transfected with a Tax expression plasmid pCMV-Tax or empty plasmid pCMV. Twenty-four hours after transfection, total cell lysates were subjected to western blot analysis with antibodies as indicated. The HIF-1 target gene *VEGF* was used as a positive control. Representative blots from two independent experiments are shown. (**e**) Ectopic expression of Tax in HeLa cells downregulates Bim and Bid expression at the transcriptional level. HeLa cells were transfected with pCMV-Tax as in (**d**). Twenty-four  hours after tansfection, Bim and Bid expression was analyzed by q-PCR. Results are representative of two independent experiments each performed in triplicate assays. (**f**) Knockdown of HIF-1*α* enhances Bim and Bid expression and enhances anti-CD95- and TRAIL-mediated apoptosis in HTLV-1-infected ATL cells. MT-2 cells were transfected with HIF-1*α* siRNA. Seventy-two hours after transfection, the protein levels of HIF-1*α*, Bim and Bid were examined by western blot analysis (left panel). Representative blots from two independent experiments are shown. HIF-1*α* siRNA-transfected MT-2 cells were treated with different concentrations of anti-APO-1 (CD95) or TRAIL (right panel) for 24 h. Apoptotic cell death was determined by DNA fragmentation. Results are representative of two independent experiments each performed in duplicate assays

The experiments above show that HTLV-1-infected cells express no or only little amounts of Bid and Bim proteins (Figure 2). We then further investigated whether the expression of Bim and Bid was downregulated at the transcriptional level by a quantitative PCR (q-PCR) analysis. Consistent with the protein expression levels, the experiment showed no or only limited levels of expression of Bid and Bim mRNA compared with the non-HTLV-1-infected Jurkat T-cell line (Figure 5a). Thus, Bid and Bim expression may be suppressed at the transcriptional level in HTLV-1-infected cells.

It has been shown that the transcription factor HIF-1 suppresses Bim and Bid expression at low O_2_ or insufficient blood supply in hypoxic cells.^26, 27, 28, 29^ We asked whether suppression of Bim and Bid expression in HTLV-1-infected cells involves a Tax-mediated increase in HIF-1*α* expression. To address this question, we first compared the expression levels of HIF-1 proteins in HTLV-1-infected and non-infected T-cell lines. Western blot analysis showed that the expression of HIF-1*α*, but not HIF-1*β*, was strongly enhanced in the HTLV-1-positive T-cell lines (Figure 5b). We next investigated whether Bim and Bid expression is downregulated by Tax-mediated upregulation of HIF-1*α* expression. Using the ERtax/ERΔtax- inducible system, expression of HIF-1*α*, but not HIF-1*β*, was shown to be enhanced upon induction of Tax by 4-HT (Figure 5c). The same results were obtained by transient transfection of a Tax expression plasmid into HeLa cells (Figure 5d). Ectopic expression of Tax in HeLa cells also led to a reduced expression of Bim and Bid at the transcriptional level (Figures 5d and e). To further investigate the role of HIF-1*α* in the regulation of Bim and Bid expression in HTLV-1-infected cells, we carried out an HIF-1*α* knockdown experiment using an siRNA approach. Knockdown of HIF-1*α* expression in MT-2 cells resulted in an increase in Bim and Bid expression and, consequently, to sensitization of MT-2 cells towards anti-CD95- and TRAIL-induced apoptotic cell death (Figure 5f). These results demonstrate that Tax-mediated overexpression of HIF-1*α* is responsible for the suppression of Bim and Bid expression.

### Targeting HIF-1*α* by chetomin restores Bim and Bid expression and enhances anti-CD95/TRAIL- and anticancer drug-induced apoptosis in HTLV-1-infected cells

**Figure 6:**
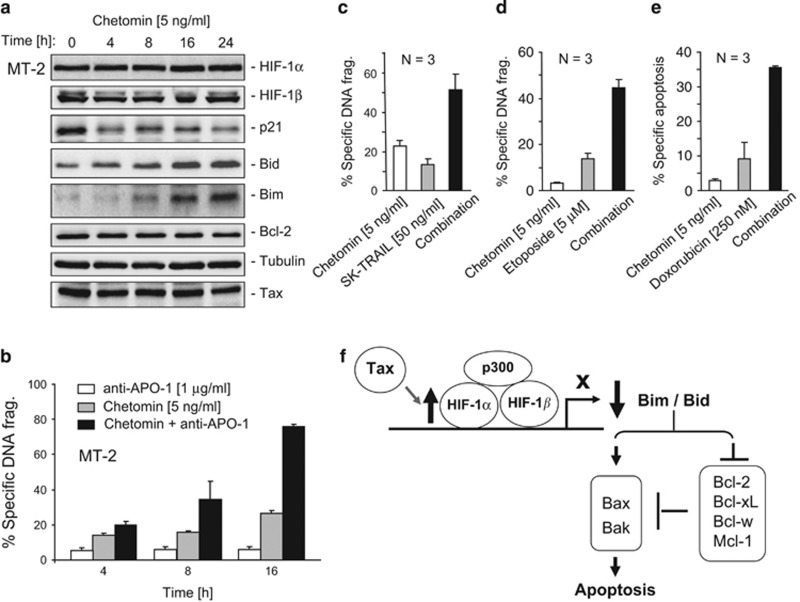
Inhibition of HIF-1*α* transactivation activity in HTLV-1-infected T cells enhances anti-CD95-, TAIL- and anticancer drug-induced cell death. (**a**) Inhibition of HIF-1*α* transactivation activity by chetomin enhances Bid and Bim expression in HTLV-1-infected T cells. MT-2 cells were treated with chetomin (5 ng/ml) for different time periods as indicated. The effect of chetomin on Bim and Bid expression was examined by western blot analysis. The HIF-1*α* target gene *p21* was used as a positive control for the inhibitory effect of chetomin on HIF-1*α*. The non-HIF-1*α* target genes *Bcl-2* and *Tax* were used as specificity controls. Representative blots from two independent experiments are shown. (**b**–**e**) Inhibition of HIF-1*α* transactivation activity by chetomin enhances sensitivity of HTLV-1-infected T cells towards anti-CD95-, TAIL- and anticancer drug-induced cell death. MT-2 cells were preincubated with chetomin (5 ng/ml) for 24 h and then treated without (solvents) or with anti-APO-1 (CD95) (1 *μ*g/ml), TRAIL (50 ng/ml), etoposide (5 *μ*M) or doxorubicin (250 nM) for 24 h. Results are representative of three independent experiments each performed in duplicate assays. (**f**) Schematic description of Tax-mediated downregulation of Bid and Bim expression via enhancing HIF-1*α* protein level and the consequent reduction in apoptosis

As HIF-1*α* was shown to have an important role in the survival of HTLV-1-infected cells, we asked whether HIF-1*α* could be a therapeutic target for treatment of Tax-expressing T cells. To address this question, chetomin, which inhibits HIF-1 transactivation activity by interfering with the interaction of HIF-1*α* with the coactivator p300,^30^ was used to treat the HTLV-1-infected T-cell lines MT-2 and MT-4. Inhibition of HIF-1*α* activity by chetomin resulted in an increase in Bim and Bid expression in MT-2 and MT-4 cells (Figure 6a and Supplementary Figure S4A). In contrast, p21, whose expression is positively regulated by HIF-1*α*,^31^ was downregulated by chetomin (Figure 6a). Tax levels were not affected by chetomin (Figure 6a). Therefore, enhanced expression of Bim and Bid by chetomin was not due to the suppression of Tax expression. Chetomin-mediated upregulation of Bim and Bid expression was shown to correlate with enhanced apoptotic cell death induced by anti-CD95, TRAIL and the anticancer drugs etoposide and doxorubicin in MT-2 and SP cells (Figures 6b–e and Supplementary Figure S4B). Thus, HIF-1*α* may be a potential therapeutic target for treatment of HTLV-1 infectious diseases.

Taken together, Bid and Bim have been shown to promote apoptosis by both inhibition of antiapoptotic Bcl-2 members and activation of Bak and Bax^16, 17, 18, 19, 20^ (Figure 6f). In this study, we show that Bid and Bim expression is suppressed in HTLV-1-infected cells through Tax-mediated elevation of the protein levels of the transcription factor HIF-1*α*. We also show that targeting HIF-1*α* may restore Bid and Bim expression and enhance chemo- and receptor-mediated apoptosis in HTLV-1-infected T cells (Figure 6f).

## Discussion

Upregulation of antiapoptotic proteins by the HTLV-1 protein Tax has been implicated in apoptosis resistance of infected cells.^6, 7, 8, 9, 10, 11^ In this study, we show that not only expression levels of antiapoptotic proteins but also proapoptotic proteins are dysregulated in HTLV-infected cells. We discovered that expression levels of the BH3-only proteins Bid and Bim are downregulated at the transcriptional level in HTLV-1-infected cells and that this downregulation is mediated by Tax. We also demonstrate that downregulation of Bid and Bim expression in HTLV-1-infected T cells has a significant impact on apoptosis resistance to anti-CD95, TRAIL and anticancer drugs (Figure 6f).

Up to now, the most successful treatment strategy for ATL patients has been reported to be a combination of the anti-viral drug Zidovudine (AZT) with interferon-*α* (IFN-*α*).^32^ However, combinational chemotherapy, the so-called VCAP-AMP-VECP: vincristine, cyclophosphamide, doxorubicin and prednisone (VCAP), doxorubicin, ranimustine and prednisone (AMP) and vindesine, etoposide, carboplatin and prednisone (VECP),^33^ achieved only a 3-year survival rate in 24% of ATL patients in Japan.^5^ We tested four HTLV-1-positive T-cell lines with regard to their responses to doxorubicin and etoposide. All cell lines showed complete (Figure 1a) or significant (Figure1b) resistance to etoposide and doxorubicin compared with the non-HTLV-1-infected leukemic T-cell line Jurkat, indicating that ATL cells are highly resistant to chemotherapy.

A systematic analysis of the expression levels of anti- and proapoptotic proteins in HTLV-1 Tax-expressing cell lines revealed that in addition to an increase in antiapoptotic proteins (Figure 1c), conversely, the expression levels of the BH3-only proteins Bim and Bid were diminished (Figure 2a). This observation was further confirmed by analyzing HTLV-1-infected CD4^+^ T cells from ATL patients (Figure 2b) and by overexpression of Tax in non-Tax-expressing cells (Figures 2c and d).

Embryo fibroblasts and T cells from Bid^−/−^ and Bim^−/−^ double-knockout mice were shown to be resistant to diverse apoptotic stimuli, for example, UV-irradiation, *γ*-irradiation, tunicamycin and etopside.^20, 33^ Thus, we predicted that reduction of Bim and Bid expression may contribute to apoptosis resistance of HTLV-1-infected T cells. To test this prediction, we carried out two experiments: ectopic expression of Bim and Bid in MT-2 and Hut-102 cells and knockdown of Bim or Bid using an siRNA approach in Jurkat and HeLa cells. Consistent with previous studies,^20, 34^ our experiments confirmed that Bim and Bid had an important role in apoptosis induction by etoposide, anti-CD95 antibody and TRAIL in leukemic T cells (Figure 3 and Supplementary Figure S1; Figure 4 and Supplementary Figure S3).

Bim and Bid not only have overlapping functions but also show selectivities.^35^ Bim interacts with Bax but not Bak, whereas Bid can interact with Bax and Bak after processing by caspases into its active form tBid. Bim binds all five prosurvival proteins, whereas tBid binds potently to Mcl-1 and Bcl-xL. Furthermore, Bak activation requires the neutralization of only Bcl-xL and Mcl-1, whereas Bax-mediated apoptosis requires the additional neutralization of Bcl-2, and probably Bcl-w. Consistent with these facts, we observed that knockdown of both Bim and Bid resulted in a further resistance to apoptosis induction compared with that seen by knockdown of either one. We show that HIF-1*α* suppresses both Bim and Bid expression (Figure 6a). Thus, HIF-1*α* may be a good therapeutic target in HTLV-1-infected cells (Figure 6f). We also observed an enhancement of Bax expression in HTLV-1-infected cell lines. Further experiments confirmed that enhanced Bax expression is regulated by Tax but not by HIF-1*α* (unpublished data).

Comparison of mRNA expression levels of Bid and Bim in HTLV-1-infected and non-infected cells revealed a substantial reduction of Bid and Bim mRNA expression in HTLV-1-infected cells (Figure 5a). As HIF-1 has been implicated in transcriptional suppression of Bim and Bid expression in several systems,^26, 27, 28, 29^ we examined the HIF-1 protein levels in HTLV-1-Tax-expressing T-cell lines. All cell lines tested showed a significant elevation of HIF-1*α* protein (Figure 5b). Using the Jurkat-ERTax/ERΔTax-inducible system, we demonstrated that Tax expression is tightly associated with the elevation of HIF-1*α* (Figure 5c). To further examine the role of Tax in the upregulation of HIF-1*α*, in general, we transiently transfected a Tax expression plasmid into HeLa cells. The experiment showed that ectopic expression of Tax in HeLa cells enhanced HIF-1*α* protein expression, which correlated with a reduction in Bim and Bid expression at the transcriptional level (Figures 5d and e). Several studies have linked the inverse correlation between HIF-1*α* and Bim/Bid expression to chemo- and radiotherapy resistance in different types of cancer.^26, 28, 29, 36, 37^ Consistent with these studies, we could show that knockdown of HIF-1*α* in MT-2 cells by siRNA led to an increase in Bim and Bid expression and rendered the cells more sensitive to anti-CD95- and TRAIL-induced apoptosis (Figures 5f and g). Thus, HIF-1*α* may have an important role in apoptosis resistance in HTLV-infected cells.

HIF-1*α* expression is regulated mainly by two distinct mechanisms: an oxygen-dependent mechanism that often occurs in hypoxic tissues or hypoxic tumor areas and an oxygen-independent mechanism through PI3K/AKT-mediated protein synthesis.^38^ PI3K is a target of Tax. Tax can bind to PI3K and stimulates the phosphorylation of AKT and, consequently, increases translation.^39^ Therefore, in HTLV-1-infected cells, Tax may increase HIF-1*α* by accelerating HIF-1*α* protein synthesis.

The tumor suppressor p53 has been shown to bind to the Bid promoter p53-responsive sequence and activate Bid transcription.^40^ However, in HTLV-1-infected cells, p53 transcriptional activity has been shown to be inhibited by Tax by the constitutive activation of NF-*κ*B (p65), resulting in the formation of a p65–p53 complex at the p53-responsive promoter sequence and blocking recruitment of important cofactors.^41, 42^ Thus, inactivation of p53 transcriptional activity by Tax may also be involved in the reduction of Bid expression in HTLV-1-infected cells. To test this possibility, we used an NF-*κ*B inhibitor to inhibit NF-*κ*B activity in SP cells. Inhibition of NF-*κ*B activity indeed showed an increase in Bid expression but at a low degree (data not shown). Thus, we conclude that Tax-mediated elevation of HIF-1*α* is the important mechanism that causes the downregulation of Bid expression.

It has been shown that Tax physically interacts with the proteasome core and enhances proteolytic activity.^43^ A recent study shows that proteasome inhibitor MG132 upregulates Bim protein levels by reducing activated ERK, a negative regulator of Bim expression.^44^ Thus, Tax-mediated increase in proteolytic activity may also be involved in the reduction of Bim expression in HTLV-1-infected cells.

HIF-1*α* is overexpressed in many types of cancer and its overexpression correlates with chemo- and radiotherapy resistance.^45, 46, 47^ Therefore, HIF-1*α* has been considered to be a therapeutic target in cancer treatment.^37^ To investigate the potential of HIF-1*α* as a therapeutic target for the treatment of HTLV-1-infected T cells, chetomin, which inhibits the HIF-1*α* transactivation activity by blocking the interaction of HIF-1*α* with its cofactor p300,^30^ was used in our experiments. The study showed that inhibition of HIF-1*α* activity by chetomin restored Bim and Bid expression in HTLV-1-infected cells (Figure 6a and Supplementary Figure S4A). Consequently, chetomin treatment significantly enhanced anti-CD95-, TRAIL-, etoposide- and doxorubicin-induced apoptotic cell death in HTLV-1-infected cells (Figures 6b–e and Supplementary Figure S4B). These results suggest that HIF-1*α* may be a potential target for the treatment of HTLV-1 infectious diseases. During the past decade, inhibitors that specifically target HIF-1*α*, for example, EZN-2968 (Enzon Pharmaceuticals Inc., Piscataway, NJ, USA and Santaris Pharma A/S, Roche Innovation Center Copenhagen A/S, Hørsholm, Denmark), an RNA antagonist that specifically targets HIF-1*α* mRNA and PX-478 (*S*-2-amino-3-[4′-*N*,*N*,-bis(2-chloroethyl)amino]phenyl propionic acid N-oxide), which inhibits HIF-1*α* protein synthesis, have been tested in various tumors and have achieved promising results *in vitro* and *in vivo* in animal tumor models.^48, 49, 50^ PX-478 and EZN-2968 are currently evaluated in phase I clinical trials. Our data may stimulate further investigations of treatment of HTLV-1-infected patients by specific HIF-1*α* inhibitors.

## Materials and Methods

### Cell lines and culture

The human malignant cell lines used in this study are the human acute T-lymphoblastic leukemia cell lines Molt-4,^51^ DND-41,^52^ CEM,^53^ Jurkat,^54^ Jurkat stably transfected with an ERtax or an ERΔtax whose expression can be induced by 4-HT,^24^ the cervical cell line HeLa^55^ and the HTLV-1-transformed human T-cell leukemia cell lines MT-2^56, 57^ and MT-4,^58^ the HTLV-1-infected ATL cell lines Hut-102^59^ and SP.^60^ All cells were cultured at 37 °C and 5% CO_2_ in RPMI-1640 or DMEM (Gibco Laboratories, Grand Island, NE, USA), supplemented with 10% FCS, 100 U/ml penicillin (Gibco), 100 *μ*g/ml streptomycin (Gibco) and 2 mM L-glutamine (Gibco). SP cells were cultured with an additional supplement of 50 U/ml IL-2 as described previously.^60^ Primary leukemic samples from HTLV-1-infected patients were kindly supplied by Charles RM Bangham (Department of Immunology, Imperial College, Wright-Fleming Institute, Norfolk Place, London, UK).

### Determination of apoptosis

Cells were treated with different concentrations of anti-APO-1 antibodies,^61^ Super*Killer*-TRAIL (Enzo Life Sciences, New York, NY, USA), etoposide (Sigma-Aldrich, St Louis, MO, USA), doxorubicin (Sigma-Aldrich) or chetomin (Enzo Life Sciences) for different time periods as indicated in the figure legends. Apoptotic cell death was examined by the analysis of DNA fragmentation according to the method of Nicoletti as described previously.^8^ Results are presented as the percent of specific DNA fragmentation using the formula: (percentage of experimental apoptosis−percentage of spontaneous apoptosis)/(100−percentage of spontaneous apoptosis) × 100.

### Western blot analysis

A total of 1 × 10^6^ cells were lysed for each sample, as described previously.^25, 62^ Equal amounts of protein were separated on 7.5–13% SDS-PAGE depending on the molecular size of the proteins of interest, blotted onto a nitrocellulose membrane (Amersham Biociences, Little Chalfon, UK) and blocked with 5% non-fat dry milk in PBS/Tween (0.05% Tween-20 in PBS). Antibodies directed against the following proteins were used: anti-c-Flip was made by our own laboratory;^63^ anti-Bad, -Bak, -Bax, -Bcl-xL, -Bcl-w, -Bid, -Bim, -HIF-1*α*, -HIF-1*β*, -Puma, -VEGF and -XIAP were purchased from Cell Signaling Technology (Danvers, MA, USA); anti-Bcl-2, -Mcl-1, -p21 and -p27 were purchased from Santa Cruz Biotechnology (Heidelberg, Germany); anti-Tax from NIH (Hybridoma, Bethesda, MD, USA; cat. no. 1312); anti-tubulin and -actin were purchased from Sigma-Aldrich (Munich, Germany).

### Quantitative real-time PCR

RNA was isolated from 1 × 10^6^ cells per preparation, using the RNeasy Mini Kit (Qiagen, Hilden, Germany) according to the manufacturer's instructions. One microgram of total RNA was reverse transcribed using the Perkin-Elmer GeneAmp RNA PCR Kit (Foster City, CA, USA). For TaqMan quantitative real-time PCR, primers and fluorescent-labeled probes for the human Bid, Bim and 18S rRNA were purchased from Sigma-Aldrich (Germany). The levels of mRNA relative to 18 S rRNA or GAPDH were calculated using the formula: relative mRNA expression=2^−(*C*_t_^ ^of Bid/Bim−*C*_t_^ ^of 18 S rRNA)^, whereas *C*_t_ is the respective threshold cycle value.

### Transient transfection studies

Two micrograms of Bid or Bim expression plasmids (Thermo Fisher Scientific, Waltham, MA, USA) were transfected into MT-2 cells using Nucleofector solution (Nucleofector Kit V, program O-17; Amaxa Biosystems, Cologne, Germany) according to the manufacturer's instructions. Forty-eight hours after transfection, cells were collected for protein expression analysis by western blot and for treatment as indicated in the figure legends. Jurkat and HeLa cells were transfected with 5 and 8 *μ*g Tax expression plasmid,^24^ respectively. Jurkat cells were transfected using electroporation with a Gene Pulser II at 250 V and 950 *μ*F (Bio-Rad, Hercules, CA, USA) and transfection of HeLa cells was carried out using Lipofectamine 2000 (Invitrogen, Paisley, UK) according to the manufacturer's instructions. Cells were collected 24–48 h after transfection for protein expression analysis.

### Knockdown studies

Jurkat cells (2 × 10^6^) were transfected in Nucleofector solution (Nucleofector Kit V, program X-01; Amaxa Biosystems) with 2 *μ*M of nonsense siRNA (Qiagen), Bid siRNA (Qiagen) or Bim siRNA (Qiagen) using the Nucleofector apparatus. MT-2 cells were transfected in Nucleofector solution (Nucleofector Kit V, program O-17; Amaxa Biosystems) with 2 *μ*M of nonsense siRNA (Qiagen) or HIF-1*α* siRNA (Qiagen). Seventy-two hours after transfection, cells were collected for protein expression analysis and different treatments as indicated in the figure legends.

## Abbreviations

HTLV-1: human T-lymphotropic virus type 1
ATL: adult T-cell leukemia
Bid: BH3-interacting domain death agonist
Bim: Bcl-2-interacting mediator of cell death
HIF-1*α*: hypoxia-inducible factor-1*α*
