# Supplementary material for: Tax contributes apoptosis resistance to HTLV-1-infected T cells via suppression of Bid and Bim expression
Source: Cell Death Dis. 2014 Dec 18;5(12):e1575–. doi: 10.1038/cddis.2014.536 (PMC4649845; doi:10.1038/cddis.2014.536)
Supplement: Supplementary Figure S1 [file cddis2014536x1.pdf]

Supplementary Fig. S1

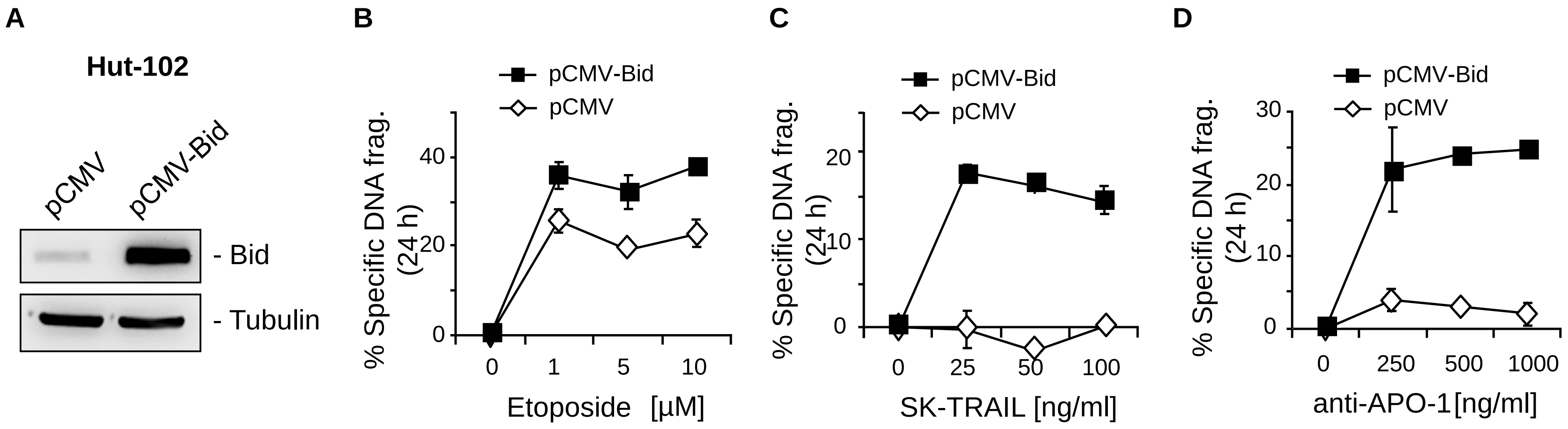

Ectopic expression of Bid in the HTLV-1-infected Hut-102 cell line enhances the sensitivity of the cells towards Etoposide-, TRAIL- and CD95-induced apoptosis.

Hut-102 cells were transiently transfected with either the empty expression plasmid pCMV or the Bid expression plasmid pCMV-Bid. Bid expression was controlled by a Western blot analysis (A). The cells were then treated with different con-centrations of Etoposide, SK-TRAIL or anti-APO-1 antibody as indicated. Apoptotic cell death was determine after 24 h Treatment by DNA fragmentation (B to D). Data are representative of two independent experiments each performed with duplicates.
