# Supplementary material for: Tax contributes apoptosis resistance to HTLV-1-infected T cells via suppression of Bid and Bim expression
Source: Cell Death Dis. 2014 Dec 18;5(12):e1575–. doi: 10.1038/cddis.2014.536 (PMC4649845; doi:10.1038/cddis.2014.536)
Supplement: Supplementary Figure S2 [file cddis2014536x2.pdf]

Supplementary Fig. S2

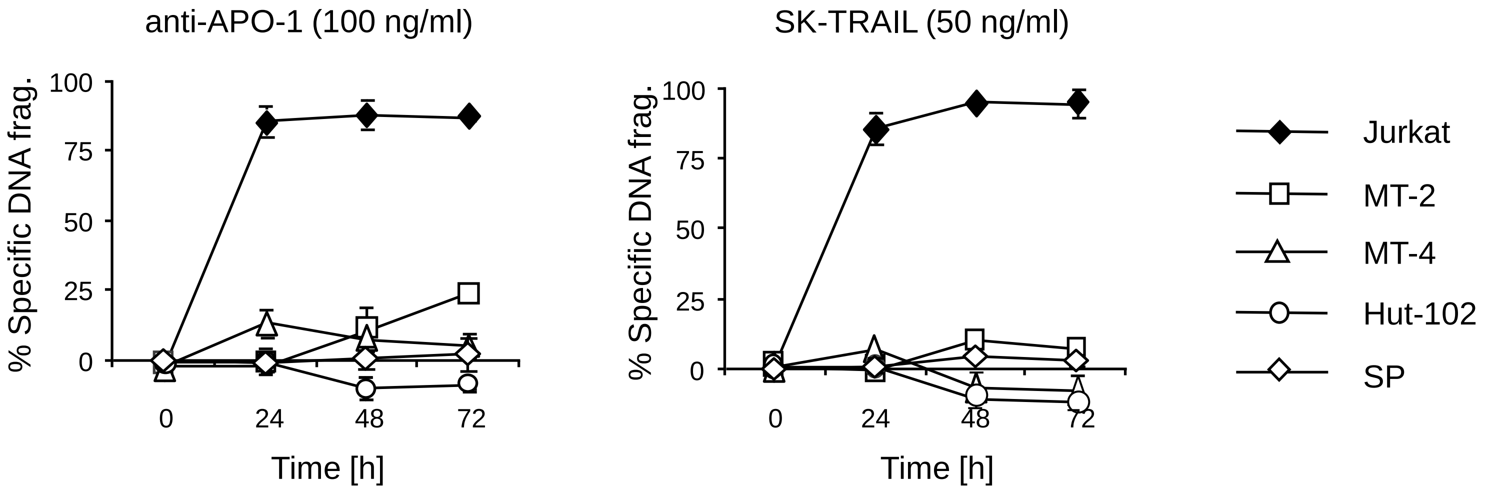

HTLV-1-infected ATL cell lines are resistant to CD95- and TRAIL-induced apoptosis.

HTLV-1-infected ATL cell lines MT-2, MT-4, Hut-102 and SP were treated with either anti-APO-1 or SK-TRAIL as indicated for different time periods. Apoptosis was determined by DNA fragmentation. Data are representatives of two independent experiments performed in duplicates.
