# Supplementary material for: Tax contributes apoptosis resistance to HTLV-1-infected T cells via suppression of Bid and Bim expression
Source: Cell Death Dis. 2014 Dec 18;5(12):e1575–. doi: 10.1038/cddis.2014.536 (PMC4649845; doi:10.1038/cddis.2014.536)
Supplement: Supplementary Figure S3 [file cddis2014536x3.pdf]

Supplementary Fig. S3

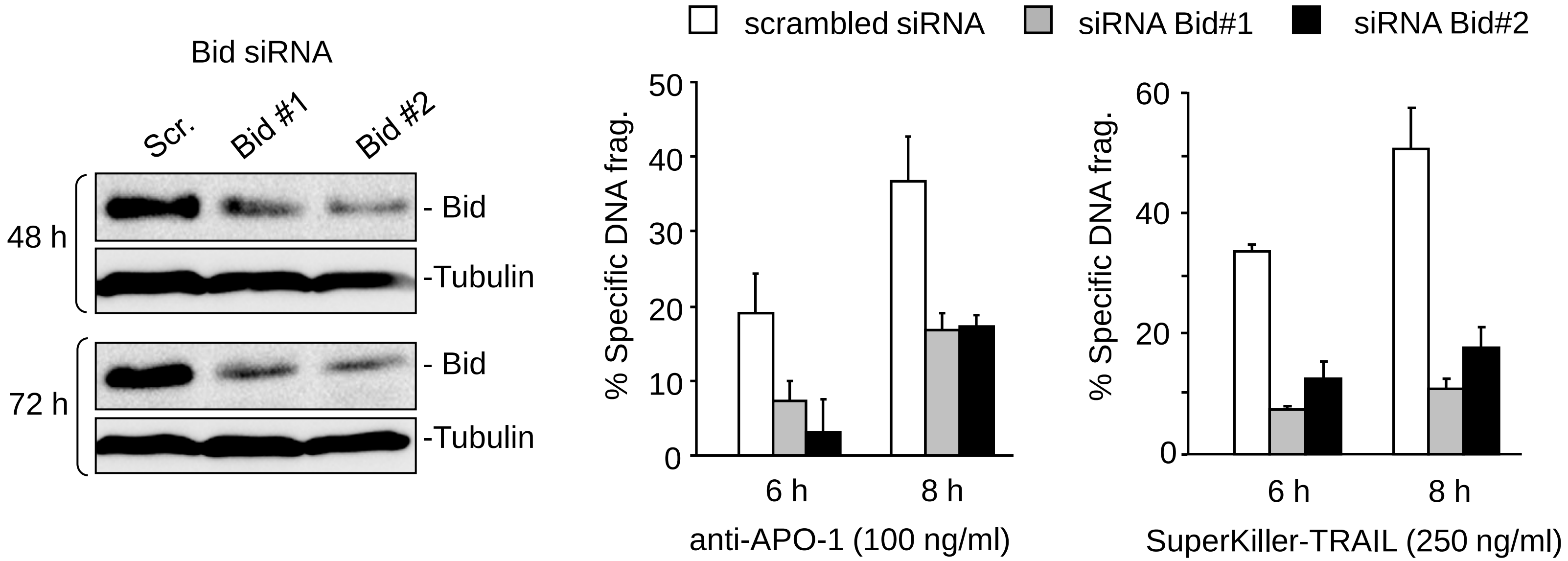

Nock-down of Bid reduces anti-APO-1- and TRAIL-mediated apoptosis in HeLa cells.

Hela cells were transfected with either the scrambled siRNA or siRNAs specific for Bid. After 48 h of transfection, cells were treated with anti-APO-1 antibody or TRAIL for the indicated times. Apoptotic cell death was determined by DNA fragmentation. Results were representative of two independent Experiments each performed in duplicates. Efficacies of nock-down were controlled by Western blot.
