# Supplementary material for: Tax contributes apoptosis resistance to HTLV-1-infected T cells via suppression of Bid and Bim expression
Source: Cell Death Dis. 2014 Dec 18;5(12):e1575–. doi: 10.1038/cddis.2014.536 (PMC4649845; doi:10.1038/cddis.2014.536)
Supplement: Supplementary Figure S4 [file cddis2014536x4.pdf]

Supplementary Fig. S4

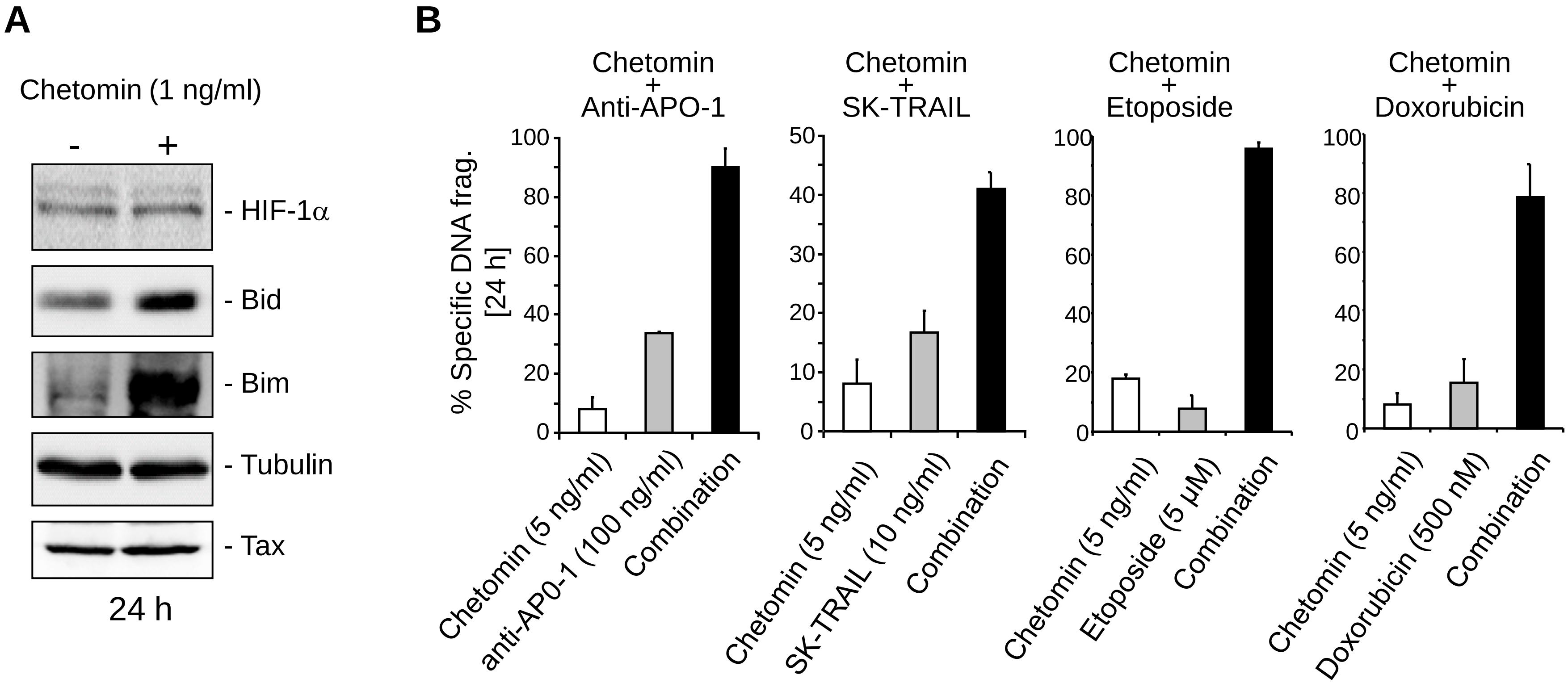

Inhibition of HIF-1α by Chetomin sensitizes anti-APO-1-, TRAIL- and anticancer drug-induced apoptosis in HTLV-1-infected cells.

(A) MT-4 cells were cultured in the absence or presence of chetomin for 24 h. The effect of Chetomin on Bid and Bim Expression was examined by Western blot with indicated antibodies. (B) Chetomin-treated SP cells were treated with different apoptosis-Inducing agents as indicated for 24 h. Apoptotic cell death was determined by DNA fragmentation. Results are representative of two independent experiments each performed in duplicate assays.
